# Multifunctional Thermal, Acoustic, and Piezoresistive Properties of In Situ-Modified Composite Aerogels with Graphene Oxide as the Main Phase

**DOI:** 10.1021/acsami.2c08042

**Published:** 2022-09-19

**Authors:** Mario Rapisarda, Michele Meo

**Affiliations:** Department of Mechanical Engineering, University of Bath, Bath BA27AY, U.K.

**Keywords:** graphene, aerogel, multifunctional, acoustic, thermal, piezoresistive

## Abstract

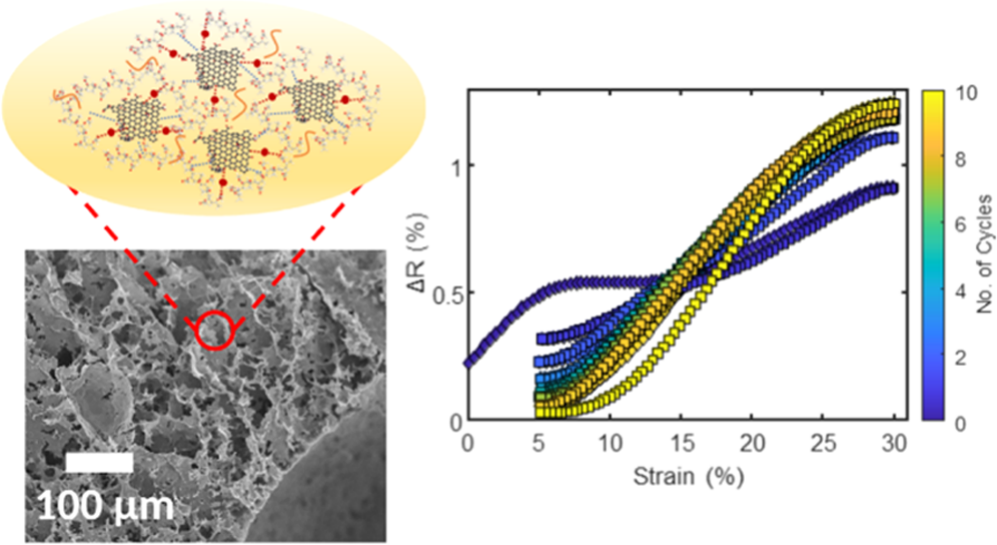

Automotive and aerospace industries require advanced materials capable of multifunctional abilities while guaranteeing limited weight and volume and simple processing. Cellular materials such as graphene-based aerogels represent a promising solution. In this study, chemical modification approaches of graphene oxide and polyvinyl alcohol (GOP) aerogels are presented. The combination of a plasticizing agent, glycerol, and a cross-linking agent, glutaraldehyde, is exploited to obtain a mechanically balanced and robust cellular structure. Modified GOP aerogels show high elastic resilience (energy loss coefficient of 29% and compressive strength of 5 kPa at 30% strain, after the 10th compression cycle), low thermal conductivity (0.0424 W mK^–1^), and high sound absorption (average coefficient of 0.72 between 500 and 1500 Hz) while maintaining a low density of 6.51 kg m^–3^ with a maximum thickness of 25 mm. Moreover, chemically reduced GOP (rGOP) aerogels are also synthesized. They are characterized by the additional feature of piezoresistive behavior, with only a marginal impact on the other properties. These results show that modified GOP and rGOP aerogels are promising candidates for the fabrication of multifunctional structures to be applied in advanced engineering applications.

## 1 Introduction

In the last decade, due to their inherent high porosity, large surface area, and low density, aerogels have been extensively studied for aerospace and automotive applications.^1,2^ In addition, advanced structures for these industries are usually required to provide multiple functionalities while respecting limited weight and size and, additionally, should derive from cheap and easy-to-process materials.^3^ A particular class of aerogels is derived from graphene oxide (GO) suspensions in water, which are able to self-assemble in three-dimensional microstructures, thanks to oxygen groups functionalizing the hexagonal lattice of carbon atoms.^4^ Although pristine GO aerogels have already been successfully used for environmental applications,^5,6^ they can further be functionalized by their combination with other chemical substances.^7^ As a result, specific properties can be tuned and optimized. For example, Wan et al. developed efficient visible-light photocatalysts by functionalizing GO with C_3_N_4_,^8^ while Lihui and co-authors incorporated GO into an alginate matrix to enhance oil/seawater separation.^9^ Moreover, oxygen functionalities can be chemically or thermally removed, making reduced GO (rGO) aerogels electrically conductive.^10^ This allowed the development of supercapacitive performance,^11^ piezoresistive properties,^12^ or electromagnetic absorption.^13^ Despite the described intriguing results, complex synthesis methods, the need for exotic chemicals, or the achievement of high performance only in highly specific conditions have, however, limited the scale-up and practical use of GO aerogels.

In our previous work, we presented an environmentally friendly manufacturing process for the fabrication of ultralight aerogels from optimized blends of GO and polyvinyl alcohol (PVA).^14^ Although very promising sound insulation performances were demonstrated, the study was lacking the inspection of other fundamental properties such as thermal stability, conductivity, and mechanical strength. From preliminary evaluations, it was found that the unbalance between bulky PVA molecules and light GO sheets, and the absence of stable cross-links between the two components was limiting the development of an organized cellular structure with high elastic resilience. Modification approaches of GO and PVA blends (GOP) were then evaluated. The enhancement due to the reduced intermolecular forces along PVA polymer chains after the addition of a plasticizer was demonstrated by Cobos et al., by adding glycerol (GLY) to chitosan/GO nanocomposites,^15^ and Hwang et al., improving the electromechanical performance, thanks to dibutyl adipate and GO inclusion in PVC gels.^16^ In addition, Zheng et al. proved cross-link formation between the hydroxyl groups present in PVA and hydroxylated carbon nanotubes using glutaraldehyde (GA) successfully.^17^ With a similar proposed mechanism, GA showed cross-linking capabilities between PVA molecules and rGO sheets for the fabrication of water-induced self-recoverable graphene aerogels for water treatment.^18^

Herein, modified GOP composite aerogels with multifunctional abilities were fabricated by ultrahigh shear mixing-promoted foaming followed by freeze-casting and freeze-drying. Two modification agents, GLY and GA, were used to enhance the cellular structure of the material. The first was chosen for its ability to relax the molecular interactions between bulky PVA molecules, while the second allows cross-link formation between GO sheets and PVA molecules. Such enhancements were confirmed by the chemicophysical characterization of GOP aerogels with varying amounts of the modification agents. Optimized amounts were chosen for their combined use, and multiple abilities of thermal and acoustic insulation and elastic resiliency were demonstrated. A chemically reduced GOP (rGOP) composite aerogel was also fabricated, which additionally proved piezoresistive properties.

Although the use of GLY to enhance the structures of the PVA composite, GA as a medium for the cross-linking of PVA or GO composite materials, and AA as a GO reducing agent was previously discussed in the literature, to the best of our knowledge, the combined and optimized use of the aforementioned agents was never considered in composite aerogels where GO represent the main phase. The novelty of the current work consists therefore in the ability to integrate the modification approach into the manufacturing process of the ultralight GO and PVA aerogels previously presented, with the only additional step of cross-linking and reduction before freeze-casting and freeze-drying. Such an approach allowed the enhancement of the cellular structure of the composite aerogel, guaranteeing lightness, mechanical robustness, acoustic and thermal insulation, and, when AA is used to promote GO reduction, piezoresistive properties.

In the Results and Discussion section, the fabrication and optimization of modified GOP aerogels are first discussed. The roles and effects of the two modification agents, GLY and GA, and the reducing agent, AA, during the manufacturing process are described and further confirmed through physicochemical characterization. The multifunctional properties of the composite aerogel are then presented in the following paragraphs, starting from physical and thermal analyses, followed by the characterization of the acoustic behavior and by the evaluation of the mechanical performance. Finally, a detailed explanation of the piezoresistive properties is discussed in the last paragraph.

## 2 Experimental Section

### 2.1 Materials

Powdered graphite oxide was purchased from Xiamen TOB New Energy, PVA pellets (98–99% hydrolyzed, medium molecular weight), GA solution (grade II, 25% in H_2_O), and AA (anhydrous) were obtained from Sigma-Aldrich, GLY (99.6%) and H_2_SO_4_ solution (0.1 M) were supplied by Fisher Scientific. Deionized MilliQ was used throughout all of the experiments. All of the chemicals were used as received without further treatment or purification.

### 2.2 Preparation of Precursors

A GO suspension in water (8 mg mL^–1^) was obtained via probe sonication (Dr. Hielscher GmbH UP100H) of GtO for 40 min under vigorous magnetic stirring and in an ice bath. PVA pellets were dissolved in water (5 wt %) through magnetic stirring at a temperature of 90 °C for 4 h. Preplasticized PVA was obtained similarly, by adding the plasticizing agent, GLY, to the PVA solution in proper amounts after 2 h of hot stirring.

### 2.3 Synthesis of Modified GO Aerogels

The modified GOP aerogels were synthesized with proper adjustments to the previously reported method of ultrahigh shear mixing and unidirectional freeze-casting of GO and PVA blends.^14^ Typically, the two components were mixed in a 1:1 ratio with low-shear magnetic stirring for 1 h, followed by 5 min of ultrahigh shear mixing (IKA Ultra-Turrax T25) at 13,000 rpm. The foamed blends were then embedded in Nomex HC cores and freeze-casted cores with the aid of silicone molds, which were placed on an aluminum heat sink submerged in liquid nitrogen. Finally, the ice-templated structures were freeze-dried (LTE LyoTrap Mini) for 96 h. Plasticized GO aerogels were obtained similarly but using preplasticized PVA with GLY contents of 5, 10, 20, and 30 wt %_GLY[PVA]_ (denoted pl_*x*_-GOP, where *x* indicates the concentration of GLY with respect to PVA weight). Cross-linked GOP aerogels were synthesized with the following additional steps: the cross-linking agent, GA, was added to the PVA solution in varying amounts of 7.5, 10, 12.5, and 15 wt %_GA[GO+PVA]_, with H_2_SO_4_ as the catalyst (6 wt %_H2SO4[GA]_); in addition, prior to freeze-casting, the foamed blends were cross-linked in an oven at 60 °C for 4 h and then cooled down to ambient temperature. The as-obtained aerogels are denoted cl_*y*_-GOP, where *y* indicates the concentration of GA with respect to GO and PVA weight. Furthermore, both the described modification approaches were combined in plasticized and cross-linked GOP aerogels: they were obtained as described for cl_*y*_-GOP, upon using the preplasticized PVA. Optimized amounts of GLY (20 wt %_GLY[PVA]_) and GA (10 wt %_GA[GO+PVA]_) were determined from a preliminary evaluation of the physicochemical properties of pl_*x*_-GOP and cl_*y*_-GOP aerogels. The sample is denoted pl_20_cl_10_-GOP. Finally, aerogels where GO was chemically reduced were manufactured as reported for pl_20_cl_10_-GOP, with the addition of the reducing agent, AA, to GO, prior to its mixing with preplasticized PVA. A 4:1 mass was chosen and 10 min of bath ultrasonication was applied to ensure an intimate dispersion. The sample was denoted rGOP, with the “pl_20_cl_10_-” appendix omitted for the sake of clarity. Modification procedures are summarized in Figure S1.

### 2.4 Physicochemical Characterization

The surface morphology and structure of the modified GOP aerogels were evaluated with scanning electron microscopy (SEM, Hitachi SU3900). Fourier transform infrared spectroscopy (FT-IR, Perkin-Elmer Frontier FT-IR spectrometer) and Raman spectroscopy (Renishaw inVia Raman microscope, 532 nm laser source) were used to investigate the chemical structure. The crystalline phases were analyzed by transmission powder X-ray diffraction (XRD, STOE STADI P, Cu Kα generator). From the latter, the interplanar distance (*d*) was calculated according to Bragg’s law (eq 1)^19^
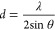
1where λ is the radiation wavelength and θ is the reflection angle of the characteristic (001) feature. The density (ρ_s_) of each sample was estimated from its weight and volume.

### 2.5 Evaluation of the Multifunctional Properties

The thermal properties of the modified aerogels were evaluated by thermogravimetric analysis (Setaram SETSYS Evolution 16 TGA/DTA) and thermal conductivity measurements (Hot Disk TPS 500 S) with the transient plane source method. The acoustic behavior was investigated by the sound absorption coefficient (α) and sound transmission loss (*STL*) measurements with a custom-built impedance tube following the standard test methods ASTM E1050 and ASTME2611,^20,21^ respectively. The mechanical performance was characterized by in-plane compression testing (Instron 3369, 100 N loadcell) with both static (0 to 70% strain with a speed of 10 mm min^–1^) and dynamic loadings (10 cycles between 5 and 30% strain at 10 mm min^–1^). For rGOP aerogels only, the electrical conductivity between the top and bottom surfaces was simultaneously measured: the connection with a digital multimeter (Keysight 34450A) was realized by two electrodes made of Cu tape and Ag conductive paste. The normalized electrical resistance (*R̅*) and the normalized electrical resistance change (), defined as expressed in eqs 2 and 3, were calculated to evaluate the piezoresistive behavior
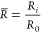
2
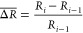
3where *R*_i_, *R*_0_, and *R*_*i*–1_ are electrical resistance values at instant *i*, at the start of the testing, and at instant *i*–1, respectively.

## 3 Results and Discussion

### 3.1 Fabrication and Optimization of Modified GOP Aerogels

**Figure 1:**
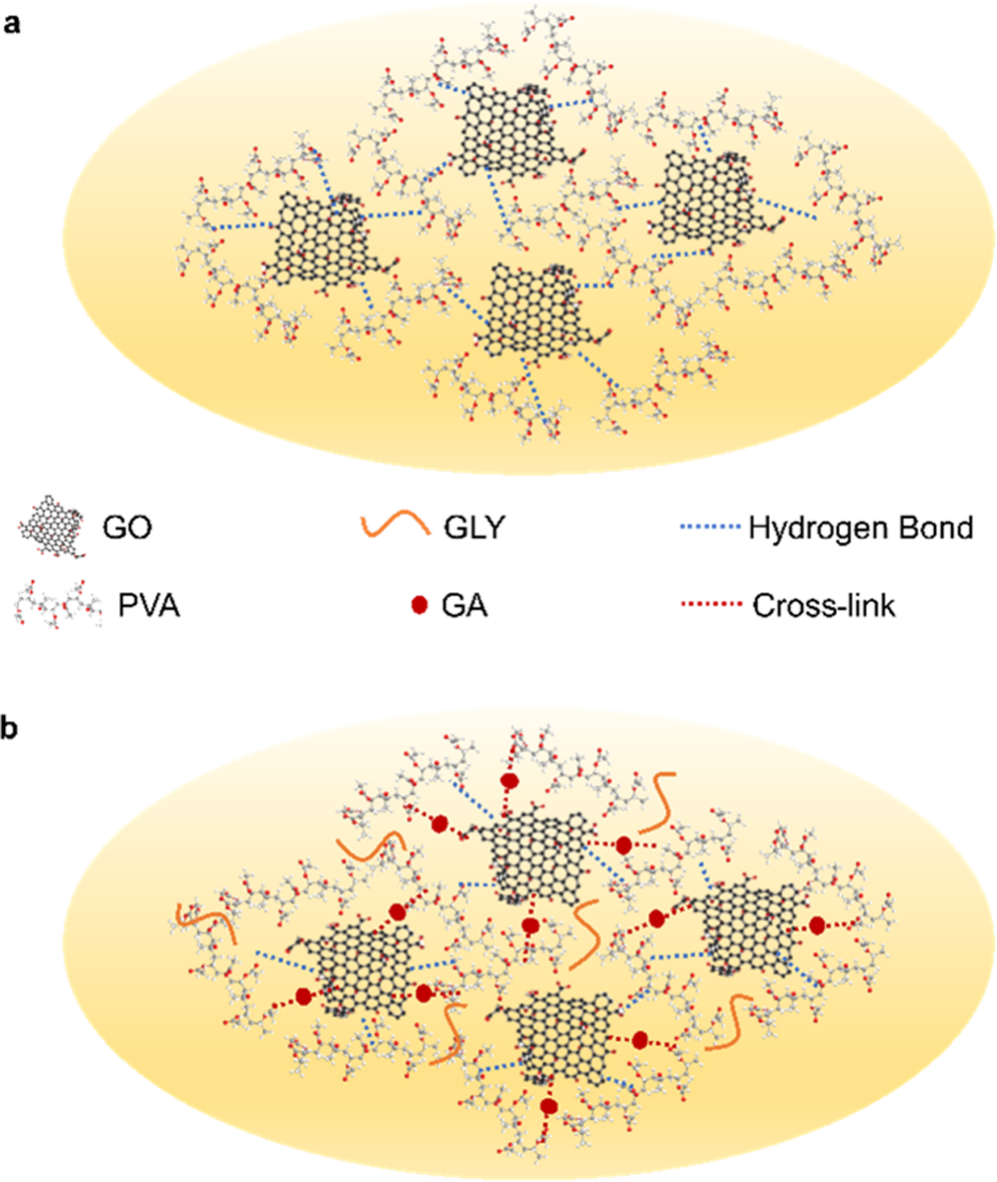
Schematic diagrams of (a) unmodified and (b) modified GOP blends.

The possibility of preparing aerogels from GO homogeneous suspensions is derived from their ability to self-assemble in 3D structures when unidirectionally frozen. As the bottom surface of the mold is exposed to a very low temperature (i.e., during the contact with the aluminum heat sink at ∼ −190 °C, thanks to the use of liquid nitrogen), small ice crystals nucleate and grow vertically, pushing the solid suspension at their boundaries and thus allowing for the formation of a templated structure. The ice can subsequently be extracted, promoting its sublimation (i.e., in drying chambers where temperature and pressure are below the triple point of water), and the remaining solid GO structure is held by van der Waals forces acting among GO sheets. With the aim to obtain aerogels satisfying the following criteria: tunable porosity and shape, lightweight yet mechanically robust, and capable of multifunctional properties, GO can be functionalized with other chemical compounds. It was previously demonstrated that PVA can be mixed with GO in homogeneous blends, thanks to hydrogen bonds between their molecules (Figure 1a). Moreover, the peculiar physical properties of such blends allow the controlled inclusion of air bubbles during an ultrahigh shear mixing step, leading to an ultralight aerogel with hierarchical porosity.^14^ To further tune the structure and improve the mechanical robustness of the aerogels, plasticizing and cross-linking agents, GLY and GA, respectively, were also introduced in the blend for this study. As schematized in Figure 1b, the two agents would affect the chemical structure of GOP with GLY, weakening the interactions between PVA molecules and GA and providing cross-linking sites between PVA molecules and GO sheets.

An optimized amount for each of the two agents was chosen for physicochemical characterization and qualitative analysis of blend processability and resulting aerogel robustness. XRD patterns in Figure S2a show the shift of the (001) crystalline phase from 10.67° of GO to 4.67° of GOP, indicating a more expanded structure due to the intercalation of PVA molecules:^22,23^ according to Bragg’s law, as reported in eq 1, the interplanar distance between GO sheets increases from 8.28 to 18.90 Å. It is also possible to observe a negligible effect of GLY inclusion in various amounts to the pl_*x*_-GOP crystalline structure. Figure S2b presents FT-IR spectra, which prove the existence of hydrogen bonds between the oxygen groups of GO and the hydroxyl functionalities of PVA.^24,25^ Moreover, it is shown that increasing amounts of GLY cause proportional weakening and shift of the C–O–C stretching of PVA around 1083 cm^–1^, thus indicating a successful interaction of the plasticizer with PVA molecules.^26^ As pictured in Figure S2c, GA inclusion in cl_*y*_-GOP has instead a marked impact on the crystalline structure. Particularly, the (101) peak of PVA disappears and the (001) feature of GO becomes sharper and shifts to lower degrees proportionally with the GA amount: from 4.67° of GOP to 3.52° of cl_7.5_-GOP, down to 3.05° for cl_15_-GOP. Both the phenomena confirm that GA effectively acts as a cross-linker between GO sheets and PVA molecules, resulting in a more expanded structure (*d* increasing from 18.90 Å of GOP to 25.07–28.93 Å, depending on the GA amount) with the PVA phase now completely amorphous.^27^ Accordingly, FT-IR spectra in Figure S2d show the formation of acetal bridges between pendant OH groups in the region between 3000 and 2800 cm^–1^ and of ether chemical bonds between 1000 and 1150 cm^–1^, proportionally as well with included GA amount.^28,29^ In terms of processability, GLY addition up to 20 wt %_GLY[PVA]_ led to an increase in the viscosity of the blend, consequently resulting in the improved stability of entrapped air bubbles. Conversely, in pl_30_-GOP, too high viscosity led to difficulties in the control of the ultrahigh shear mixing. Starting from 10 wt %_GLY[PVA]_, also the mechanical robustness improved with a marked reduction of the brittle behavior, characterizing the unmodified GOP aerogel. For cl_*y*_-GOP blends, the foaming process was negatively affected (i.e., decreased stability of entrapped air bubbles) by GA inclusion already in the smallest amount of 7.5 wt %_GA[GO+PVA]_ and proportionally worsened for higher amounts until no air bubbles could be entrapped in cl_15_-GOP. Besides, the mechanical robustness was highly improved with a remarkable elastic behavior for concentrations of GA up to 10 wt %_GA[GO+PVA]_, while higher concentrations of GA led to an excessively bulky and brittle structure.

**Figure 2:**
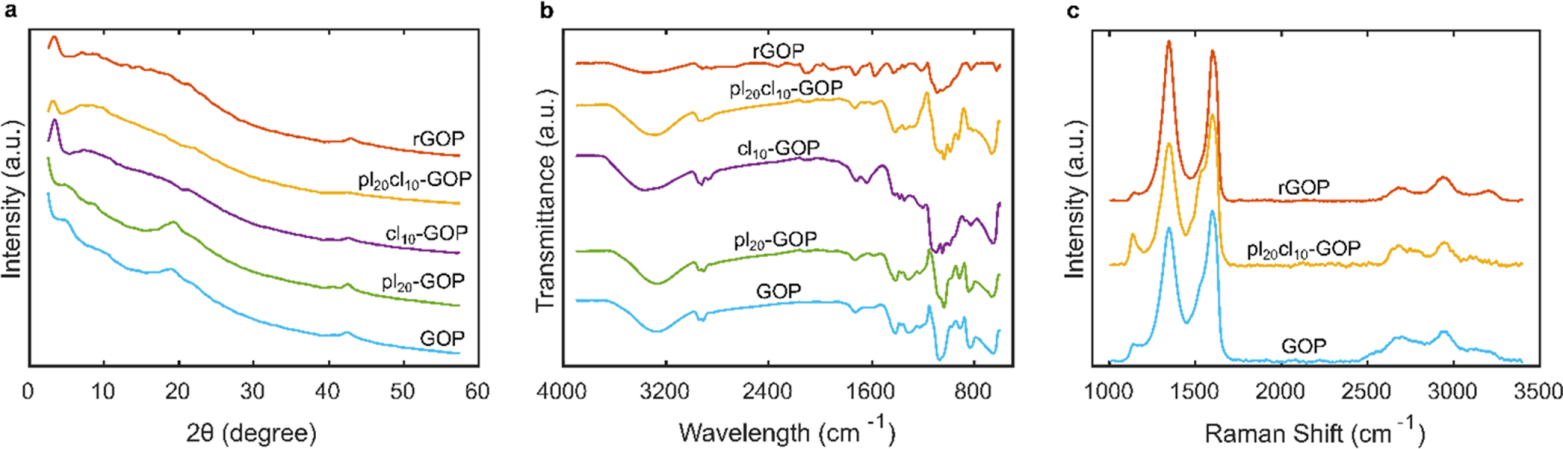
(a) XRD patterns, (b) FT-IR spectra, and (c) Raman spectra.

Due to the aforementioned reasons, the amounts of 20 wt %_GLY[PVA]_ and 10 wt %_GA[GO+PVA]_ were chosen as optimum values for the manufacturing of pl_20_cl_10_-GOP aerogels where the two modification approaches were combined. The latter blend composition was also used for the fabrication of modified aerogels with improved electrical properties, thanks to the reduction of GO promoted by the use of AA as a reducing agent. From XRD patterns in Figure 2a, it is possible to observe a further expansion of the crystalline structure from 26.03 Å (3.39°) of cl_10_-GOP to 28.19 Å (3.13°) of pl_20_cl_10_-GOP, while after the reduction, rGOP shows only a slight restacking to 26.82 Å (3.29°). Figure 2b presents the FT-IR spectra where the presence of both GLY interaction with PVA molecules and ether cross-links between GO sheets and PVA molecules promoted by GA is confirmed. In the rGOP spectrum, it is possible to observe the reduction of oxygen-related features such as hydroxyl group vibration and deformation at around 3400 and 1413 cm^–1^, respectively, and alkoxide, epoxide, and peroxide group vibrations between 1050 and 900 cm^–1^.^30,31^ Moreover, a new band is visible at 1576 cm^–1^, which is attributable to the aromatic C=C stretching and thus to the restoration of the sp^2^ lattice.^32^ Raman spectra in Figure 2b were collected to obtain more insights into GO reduction and its effects on the carbonaceous structure, while no significant differences were observed upon addition of GLY and GA to the unmodified GOP. In particular, pristine GO shows two main bands at 1352 cm^–1^ (D) and 1606 cm^–1^ (G) and secondary scattering between 2680 and 3200 cm^–1^ (Figure S2e). The first two are associated with the A_1g_ breathing mode caused by the structural disorder and the E_2g_ vibrating mode of ordered graphite crystallites.^33^ They can be quantitatively analyzed and compared with the calculation of the ratio of their intensities, the *I*_*D*_/*I*_*G*_ ratio, which is an index of the structural disorder.^34^ Three additional interbands can also be considered for a more exact deconvolution of the signal: D* at ∼1140 cm^–1^, associated with sp^2^–sp^3^ bonds at the edge of the hexagonal lattice of carbon atoms, *D*″ at ∼1540 cm^–1^, due to amorphous regions with interstitial defects, and *D*′ at ∼1620 cm^–1^, a phonon mode caused by crystal defects.^35^ All of the previously described features are observed in both unmodified GOP and in pl_20_cl_10_-GOP aerogels, without significant differences. PVA main features, which are shown at 1440 and 2914 cm^–1^ (Figure S2e), are absent in all of the aerogels, explainable by the amorphous nature of the PVA intercalated phase as well as by the much higher intensity of the signal generated by the carbonaceous structure.^36^ It is crucial to observe the behavior of the *I*_D_/*I*_G_ ratio, which is equal to 0.88 in both GOP and pl_20_cl_10_-GOP but sharply increases to 1.03 in rGOP. The latter is a clear sign of a successful reduction of GO to rGO during which the oxygen functionalities are removed by their reaction with AA, leading to a partially increased disorder in the structure but most importantly to the restoration of the hexagonal lattice of sp^2^ carbon atoms, which will be responsible for the improved electrical properties.^37^

### 3.2 Physical and Thermal Properties

**Figure 3:**
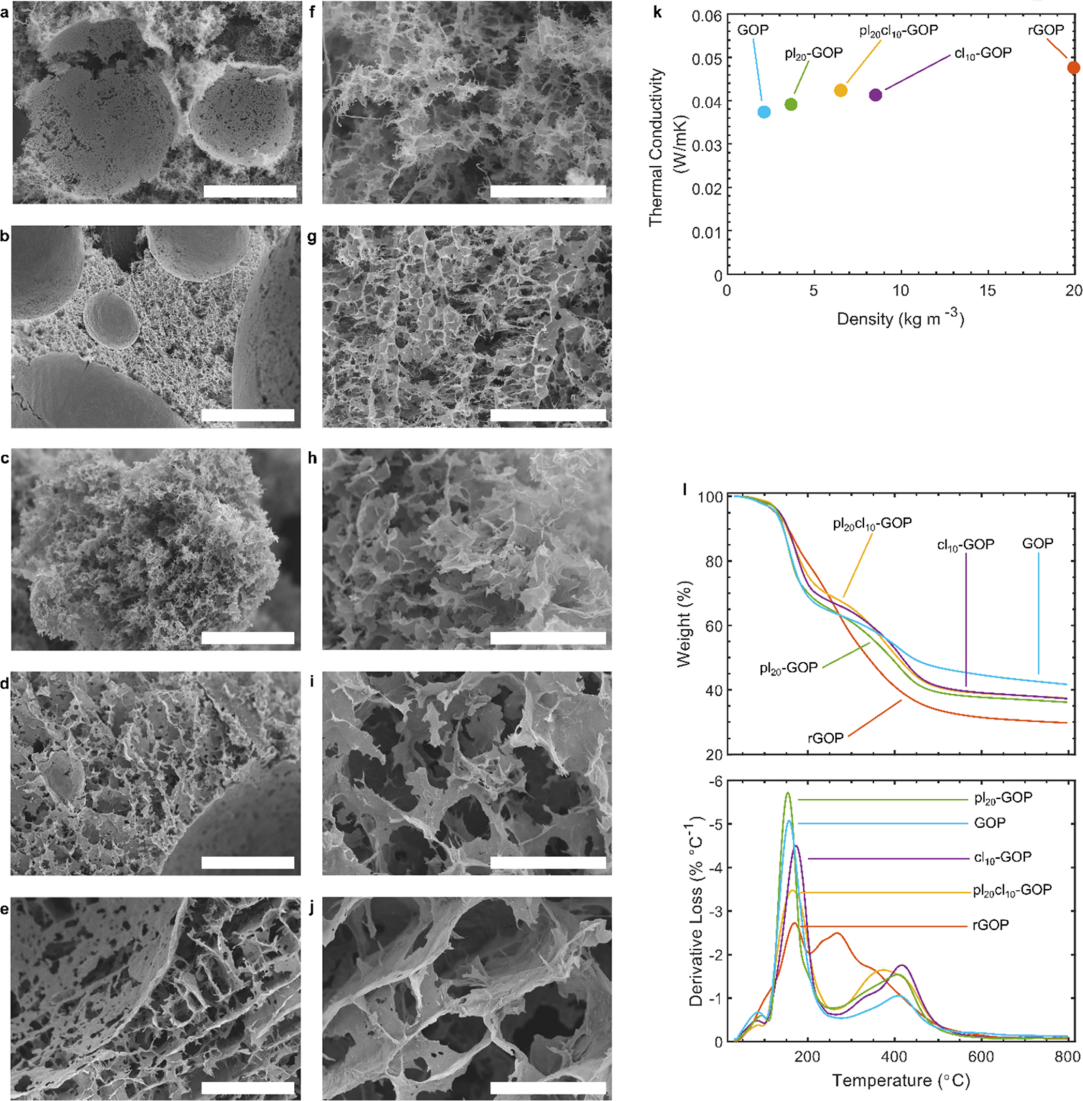
SEM imaging of (a, f) GO, (b, g) pl_20_-GOP, (c, h) cl_10_-GOP, (d, i) pl_20_cl_10_-GOP, and (e, j) rGOP. Magnifications: (a–e) ×200 (200 μm scale bar) and (f–j) ×1000 (50 μm scale bar). (k) Variation of thermal conductivity and density with chemical composition. (l) TGA and dTGA curves.

The surface morphology of cryogenically fractured aerogels is pictured in Figure 3. From low-magnification images (Figure 3a–e), the hierarchical porosity derived from the inclusion of air bubbles during the ultrahigh shear mixing is visible for all samples, although with some differences. Particularly, the stabilizing effect of GLY and the detrimental one of GA on air bubbles during the processing are highlighted in Figure 3b,c, respectively. When both agents are included in pl_20_cl_10_-GOP, the two opposing effects appear balanced (Figure 3d), while no differences with respect to the latter can be appreciated in rGOP (Figure 3e). By observing higher magnification images (Figure 3f–i), it is found that while GLY inclusion leads to a more ordered pore geometry and more defined walls with respect to unmodified GOP (Figure 3f–g), the cross-links in cl_10_-GOP result in a bulkier structure with less interconnection between the pores (Figure 3h). The presence of both agents leads to an interconnected porous structure, although with bigger pores with respect to GOP or pl_20_-GOP (Figure 3i). Figure 3j shows that pl_20_cl_10_-GOP and rGOP structures are similar, but with thicker pore walls for the latter.

The variation of the physicochemical structure of modified aerogels directly affects their density and thermal conductivity (Figure 3k). Particularly, the unmodified GOP shows the lowest values for both: 2.10 kg m^–3^ and 0.0374 W mK^–1^, respectively. The addition of 20 wt %_GLY[PVA]_ or 10 wt %_GA[GO+PVA]_ causes an increase in both properties, with a bigger impact from GA (8.51 kg m^–3^ and 0.0413 W mK^–1^, respectively). Interestingly when combined in pl_20_cl_10_-GOP, the modifying agents lead to an intermediate density of 6.51 kg m^–3^, thanks to the increased porosity, while the thermal conductivity reaches 0.0424 W mK^–1^, explainable with the cross-linked pore walls. GO reduction severely affected both density and thermal conductivity, showing values of 19.92 kg m^–3^ and 0.0479 W mK^–1^, respectively. The first is due to a reduced ability of the blend to stabilize air bubbles after the addition of AA, while the second is derived from the partial restoration of the carbon lattice after the removal of oxygen functionalities.

The thermal stability of the aerogels was also evaluated with TGA and derivative analysis (dTGA). As pictured in Figure S3, the thermal reduction of GO in GOP starts at a lower temperature (156 °C) with respect to pure GO (184 °C) but at a slower rate; PVA degradation is instead almost hindered and shifted from 268 °C (sharp peak) to ∼330–416 °C (broad peak, due to the merging with other thermal events). GLY addition causes a shift of both GO reduction and PVA degradation to slightly lower temperatures and higher rates, proportionally with its amount (Figure S3a). In cl_*y*_-GOP, the cross-linking has the same average effect of slightly delaying GO reduction and increasing the PVA degradation rate (Figure S3b). The addition of both modification agents has a positive effect on thermal stability up to 300 °C (GO reduction region), while PVA degradation starts at a lower temperature (Figure 3l). These results are in accordance with previous observations of GLY molecules, weakening the PVA chemical structure, and GA, forming cross-links between oxygen functionalities of GO and PVA. Due to the previous chemical reduction, oxygen groups of GO were already partially stripped, and thus, the thermal reduction of GO during rGOP thermal analysis had a much smaller rate. For the same reason, above 200 °C, the almost complete removal of oxygen groups, which are the sites for GO interactions and cross-links with PVA molecules, led to more severe degradation.

### 3.3 Acoustic Properties

**Figure 4:**
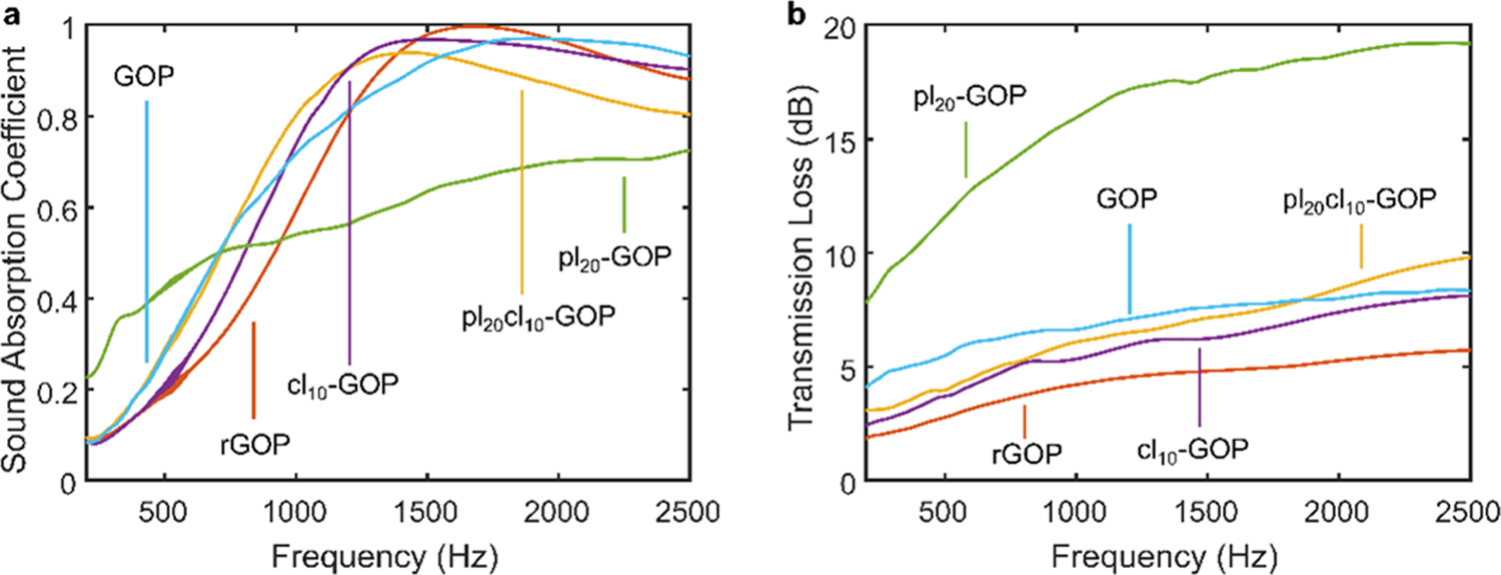
(a) Sound absorption coefficient and (b) sound transmission loss.

The acoustic properties of modified GOP aerogels with a thickness of 25 mm are presented in Figure 4, where the effects of the different physicochemical structures are clearly visible in the variation of both α and *STL* with the frequency. The higher plasticity of pl_20_-GOP causes a severe drop in sound absorption abilities above 500 Hz, while the increased stiffness due to cross-linking in cl_10_-GOP is reflected in a shift of the absorption peak to lower frequencies, at the cost of a 10% reduction of α (with respect to the unmodified GOP) in the low-frequency region between 500 and 800 Hz. The combined effects of GLY and GA in pl_20_cl_10_-GOP lead instead to the best absorption performance up to 1500 Hz. The structural changes during GO reduction determine a shift of the rGOP absorption peak to higher frequencies and optimal behavior in the mid-high frequency range between 1000 and 2500 Hz. For a direct comparison of the applicability of the modified aerogels as absorbing acoustic materials, an average sound absorption coefficient (α̅) in the low-mid range between 500 and 1500 Hz was calculated. The best result of 0.72 was achieved by pl_20_cl_10_-GOP, immediately followed by the unmodified GOP with 0.68. pl_20_-GOP and rGOP are the samples suffering most of the structural modifications, with values of 0.54 and 0.59, respectively.

Transmission losses were similarly evaluated through an averaged coefficient in the 500–1500 Hz range (). Considering the relation between α and the reflection coefficient (*R*), α = 1–|*R*|^2^, and that higher transmission losses can be predicted from a structure showing higher reflections,^38^ the best performance is expectedly found in pl_20_-GOP with a very high  value of 15.54 dB. The values for the other samples fall instead in a range between 4.07 dB (rGOP) and 6.71 dB (GOP). The described acoustic behavior can be explained by the cross-sectional distribution of large and small pores within the aerogels, which affects the damping properties of the different structures and in turn sound attenuation through the material.^39^ Correspondingly, the numerical simulation conducted by Xie et al. suggested that slow-sound propagation could be achieved in subwavelength composite aerogels with inhomogeneous structures.^40^ These results fit well with the described physical structure and acoustic behavior of modified GOP aerogels, with particular evidence in the effects of the addition of the plasticizing agent seen in pl_20_-GOP.

### 3.4 Mechanical Properties

**Figure 5:**
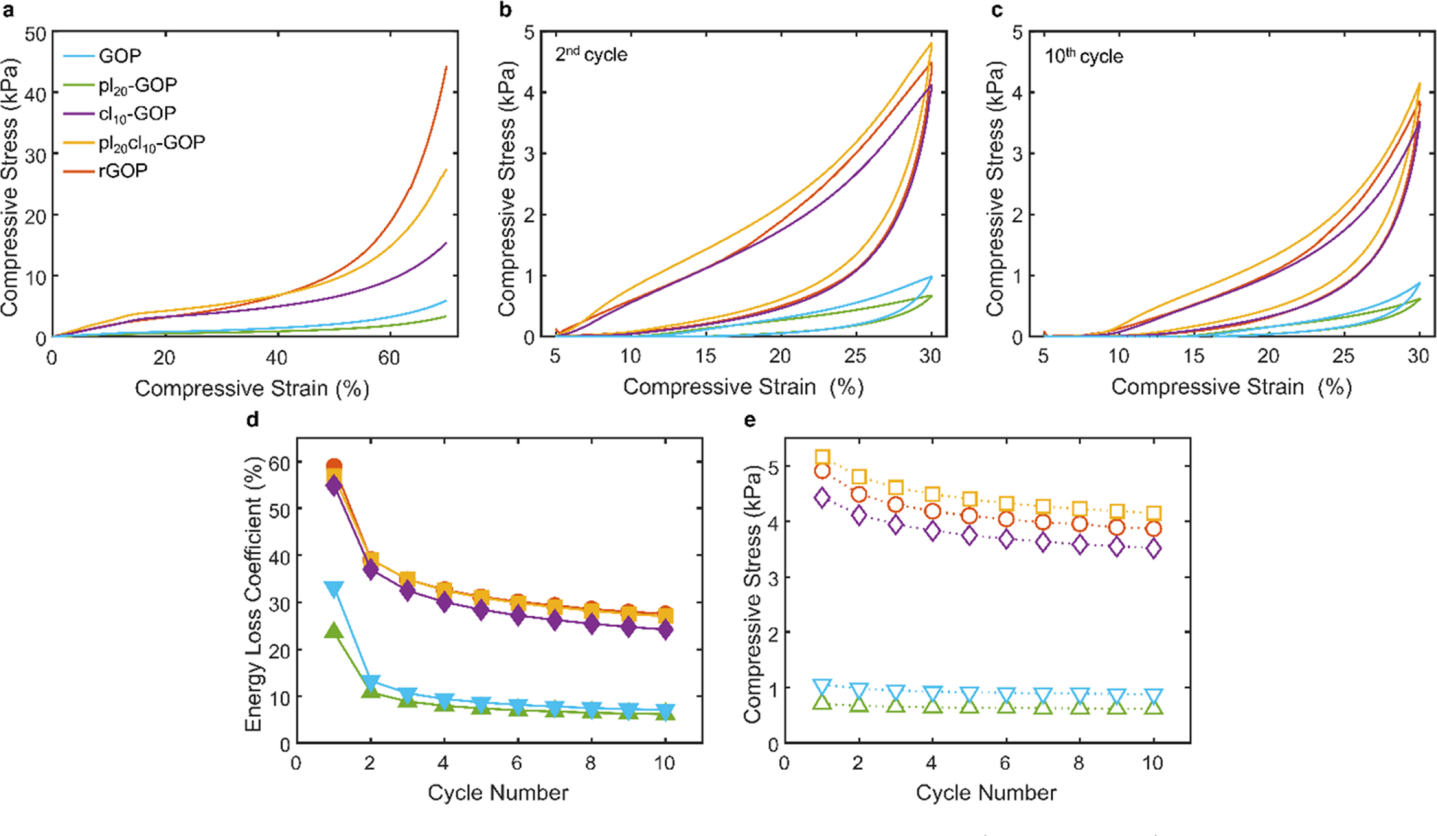
(a) Static compression loading from 0 to 75% strain, (b) 2nd and (c) 10th cycles of the dynamic compression loading between 5 and 30% strain. (d, e) Trends of the energy loss coefficient and the maximum compression stress with cycle number. The same legend applies to all of the panels.

The mechanical properties of the manufactured aerogels were quantified through static in-plane compression tests up to 70% strain. Three different regions can be distinguished in the stress–strain curves pictured in Figure 5a: an elastic region for strain below 20%, a plateau region for strain between 20 and 50%, and a densification region for strain above 50%. Moreover, no signs of structural failure can be observed. The reduction of the compressive strength with respect to the unmodified GOP reference is found in pl_20_-GOP, as a consequence of the plasticizing agent interaction with PVA molecules. Conversely, cross-links created between GO and PVA, thanks to GA addition, allow for a marked improvement of cl_10_-GOP compressive performance. Both agents synergically determine a further improvement in pl_20_cl_10_-GOP mechanical strength. A different behavior is instead seen in rGOP, where the partial loss of cross-links due to GO reduction leads to the reduced elastic and plateau region, now up to 40% of strain, whereas the bulkier structure is responsible for the highest compression strength in the densification region.

To further evaluate the structural robustness of the modified aerogels, dynamic testing with 10 loading–unloading cycles of in-plane compression up to 30% strain were performed, with the 2nd and 10th cycles pictured in Figure 5b,c, respectively. Hysteresis loops can be observed, indicating a resilient nature of the aerogels with energy dissipation due to the buckling of pore walls, friction and adhesion between the molecules, and formation of microfractures in the first cycles. These phenomena can be analyzed with the variation of the energy loss coefficient and maximum compression strength with cycle number (Figure 5d–e). All of the aerogels show a similar behavior with the energy loss decreasing in the first four cycles, before reaching more stable values. The absence of cross-links and the structural integrity entrusted only to the secondary π–π interactions between GO sheets and hydrogen bonds between GO and PVA are responsible for the low resiliency and low maximum compression strength of unmodified GOP, with GLY plasticizing effects on PVA molecules causing a further worsening. Nevertheless, the formation of cross-links promoted by GA dramatically improves both properties in cl_10_-GOP, with a further enhancement after the combined modifications in pl_20_cl_10_-GOP. Accordingly, it is possible to speculate that plasticizing molecules weaken the PVA stiff nature and enable GO sheets and thus pore walls to bend and buckle more freely under compression. For the same reason, rGOP resiliency is not affected, although the partial loss of cross-links determines a slight reduction of the compression strength in the plateau region.

### 3.5 rGOP Aerogel Piezoresistive Properties

**Figure 6:**
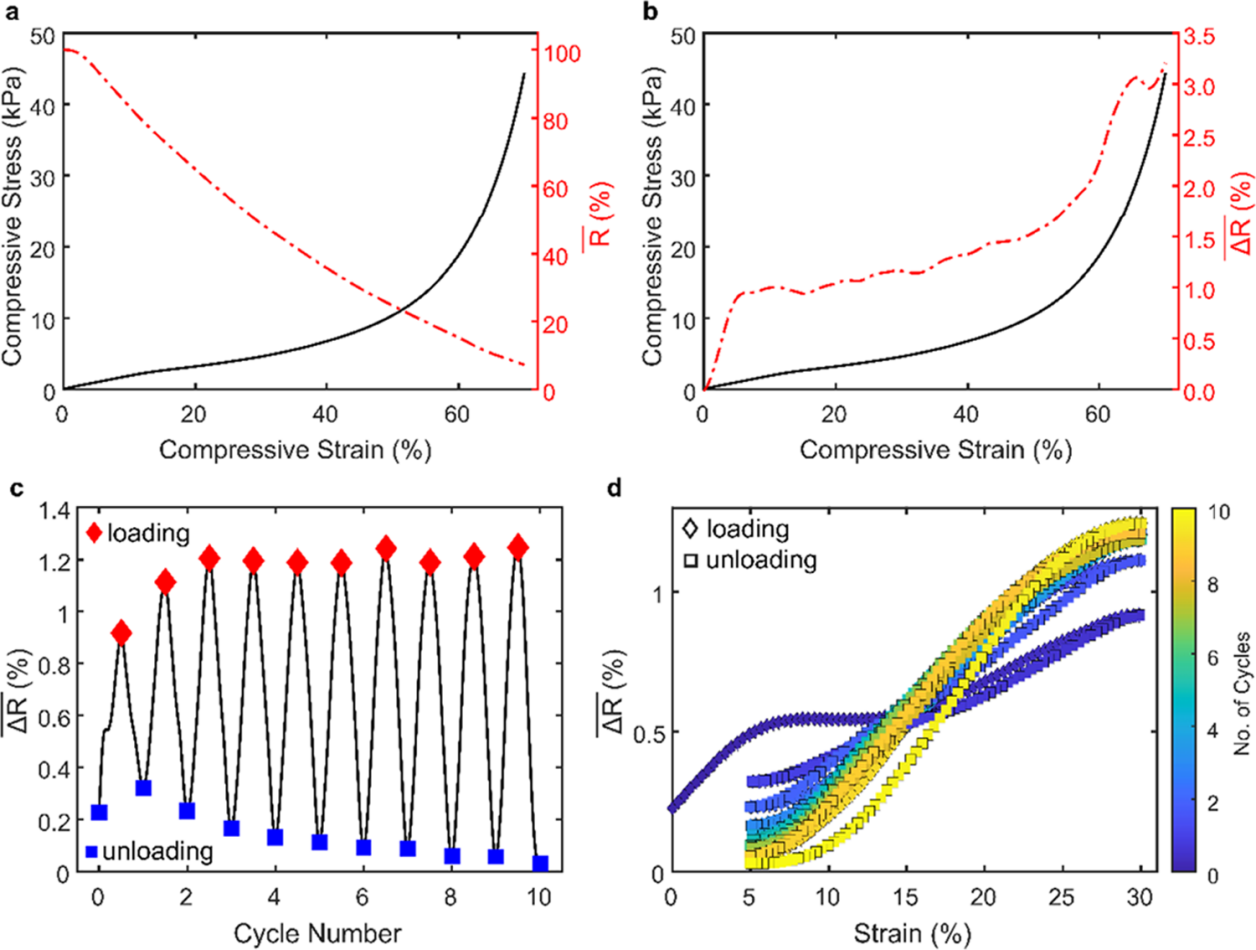
(a) Stress–strain curve and the trend of the normalized electrical resistance (*R*/*R*_0_) and (b) relationship between the normalized electrical resistance change (Δ*R*/R_0_) and the strain during static compression testing. (c) Trend of Δ*R*/*R*_0_ during cyclic compression testing. (d) Relationship between Δ*R*/*R*_0_ and the strain during cyclic compression testing.

The partial restoration of the hexagonal lattice of sp^2^ carbon atoms occurring during GO chemical reduction is responsible for improved electrical properties in rGOP aerogels. Particularly, by monitoring the electrical resistance during compression tests, it was possible to observe an intriguing piezoresistive behavior. Two parameters, *R̅* and , were analyzed for this purpose (see Experimental Section for more information). The trend of *R̅* compared to the stress–strain curve during the static testing is shown in Figure 6a, with a 92.3% decrease at 70% of strain. This is due to the densification of the structure (i.e., reduction of distance between rGO sheets) and the formation of new contact points between pore walls. Moreover, a linear relationship between  and strain is found in the elastic and plateau region, after which the trend becomes exponential. The initial steep variation is due to an increase in the contact area of the electrodes, while the anomalous variation of around 65% strain can be attributed to a densification phenomenon. During dynamic testing, the small variation of the  value further confirms the resilience of the structures of rGOP aerogels (Figure 6c). Additionally, from Figure 6d, it is interesting to observe how after the first cycles,  can precisely track the cycling of the applied strain.

## 4 Conclusions

In this work, an in situ modification approach for graphene aerogels obtained from foamed blends of GO and PVA was proposed. The aim was to keep and, eventually, tune the main features of the reference aerogel (GOP), such as the lightweight, hierarchical porosity, sound, and thermal insulating abilities while improving the mechanical robustness, and as shown for rGOP, extend the functionalities, thanks to the piezoresistive behavior. Two modification agents were adopted, a plasticizer (GLY) and a cross-linker (GA). Their effects on the physicochemical structure, due to the interaction with and between rGO sheets and PVA molecules, were studied and optimized. Both agents were introduced to the blends through specifically designed additional steps that did not affect the processability (i.e., ability to include and stabilize air bubbles during ultrahigh shear mixing) and the fabrication process in general (i.e., possibility to embed the aerogel in structural HC cores). Thanks to this approach, the pl_20_cl_10_-GOP aerogel benefitted from the modifications in terms of sound absorption abilities in the critical range of 500–1500 Hz, showing an increased average absorption coefficient of 0.72 while keeping a very low thermal conductivity of 0.0424 W mK^–1^. Most importantly, the optimized sample showed a 3-fold improvement of the compressive strength and the energy loss coefficient during static and dynamic mechanical testing, respectively. Even though the modification causes an increase in the density of the material, now reaching 6.51 kg m^–3^, the presented pl_20_cl_10_-GOP aerogel is still one of the lightest ever reported in the literature and yet is able to be used as a robust sound and heat insulator within structural HC cores (Table S1).^14,17,41−50^

**Figure 7:**
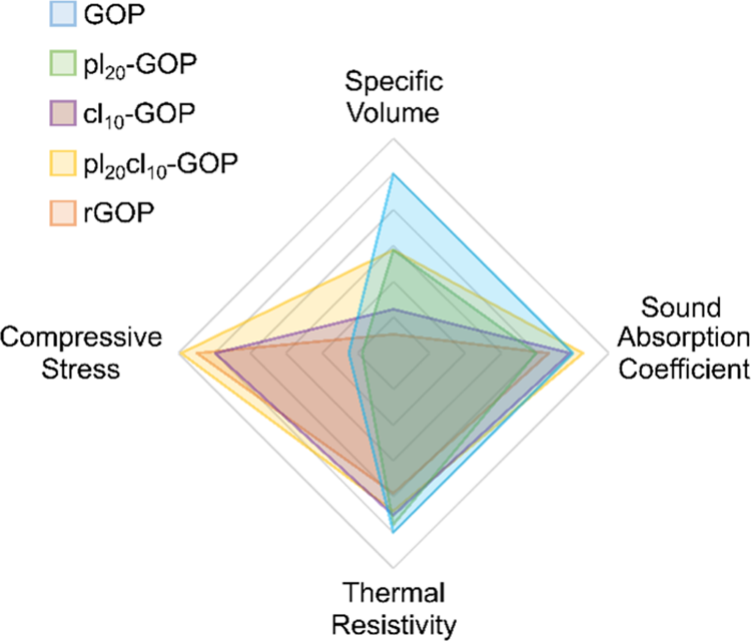
Comparison of the multifunctional properties of the composite aerogels presented in the current work. The specific volume is calculated as the inverse of the density, the average value in the 500–1500 Hz range is taken for the sound absorption coefficient, the thermal resistivity is obtained from the inverse of the thermal conductivity, and the compressive stress is taken at 30% strain of the 10th mechanical compression cycle. All values are proportionally scaled using the unmodified GOP as a reference.

It is crucial to mention that the adopted modification approach also allows the tuning of the final properties of aerogels for a specific application, with two examples that can be given from this research work. The first one is the pl_20_-GOP aerogel where the weakening action of GLY molecules to the PVA stiff phase leads to the assembly of a more ordered pores geometry and plastic mechanical behavior. This was reflected in the sound transmission abilities, where an extremely high  value of 15.54 dB and a maximum of almost 20 dB at 2500 Hz were recorded, thanks to the improved damping properties. Contemporary, the density and the thermal conductivity of the assembled aerogel (3.65 kg m^–3^ and 0.0391 W mK^–1^, respectively) only marginally increased with respect to the unmodified reference. The as-described features render the pl_20_-GOP aerogel an ideal candidate for applications where high transmission loss and thermal insulation are desired while maintaining an extremely low weight. Slightly different is the case of the rGOP aerogel, where the chemical reduction of GO obtained through the addition of AA as a reducing agent caused substantial changes in the physicochemical structure and, consequently, the properties. The most important consequence of the loss of oxygen functionalities in GO sheets and, therefore, of the partial restoration of the hexagonal lattice of carbon atoms was undoubtedly the unlocked ability of electrical conductivity. This in turn allowed for the piezoresistive properties of the rGOP aerogel: the shortening/elongation of the distance between rGO sheets and the formation/clearing of new contact points between pore walls led to the possibility of tracking the strain status during the compression/release phase of the mechanical testing through the variation of the measured electrical resistance between the electrodes. Nevertheless, the same aerogel is capable of remarking sound absorption abilities in the mid-frequency range (i.e., α never below 0.9 between 1250 and 2500 Hz) and low thermal conductivity (0.0479 W mK^–1^). A comparison of the multifunctional properties between presented composite aerogels is pictured in Figure 7.

Although further studies will be conducted to improve the mechanical robustness for a higher number of compression cycles and limit the increase in density, the presented modification method of GO and PVA blends allows the fabrication of multifunctional GOP aerogels that can be employed in aerospace, automotive, and marine transport, as well as in building and construction.

## Supporting Information Available

GOP aerogel manufacturing scheme; physicochemical characterization of modified GOP aerogels (XRD patterns and FT-IR spectra of pl*_x_*-GOP and cl*_y_*-GOP with pure GO and PVA as a reference and Raman spectra of unmodified GOP with pure GO and PVA as a reference); thermal stability of modified GOP aerogels (TGA and dTGA of pl*_x_*-GOP and cl*_y_*-GOP aerogels); and comparison of density, porosity, sound absorption, and thermal conductivity between modified GOP aerogels from this work and other aerogels with comparable thicknesses previously reported in the literature (PDF)
