# Supplementary material for: Multifunctional Thermal, Acoustic, and Piezoresistive Properties of In Situ-Modified Composite Aerogels with Graphene Oxide as the Main Phase
Source: ACS Appl Mater Interfaces. 2022 Sep 19;14(38):43646–55. doi: 10.1021/acsami.2c08042 (PMC9523609; doi:10.1021/acsami.2c08042)
Supplement: Supplementary file 1 — am2c08042_si_001.pdf [file am2c08042_si_001.pdf]

Supporting Information

Multifunctional Thermal, Acoustic and Piezoresistive Properties of In Situ Modified Composite Aerogels with Graphene Oxide as the Main Phase

Mario Rapisarda[a], Michele Meo[a,*]

[a]Department of Mechanical Engineering, University of Bath, Bath BA27AY, UK
[*]Email: m.meo@bath.ac.uk

1. Supporting figures

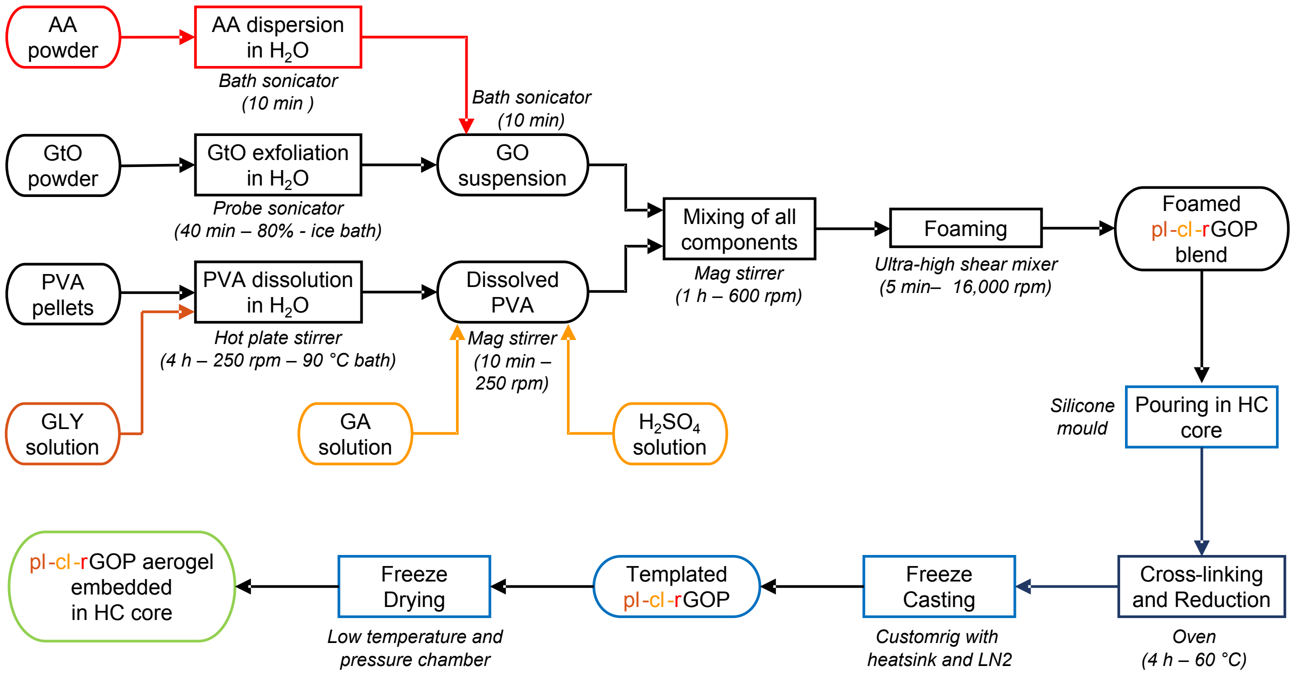

Figure S1. GOP aerogel manufacturing scheme. Modifications to the reference GOP can be distinguished by colors: plasticiser in orange, cross-linker in yellow and reducing agent in red.

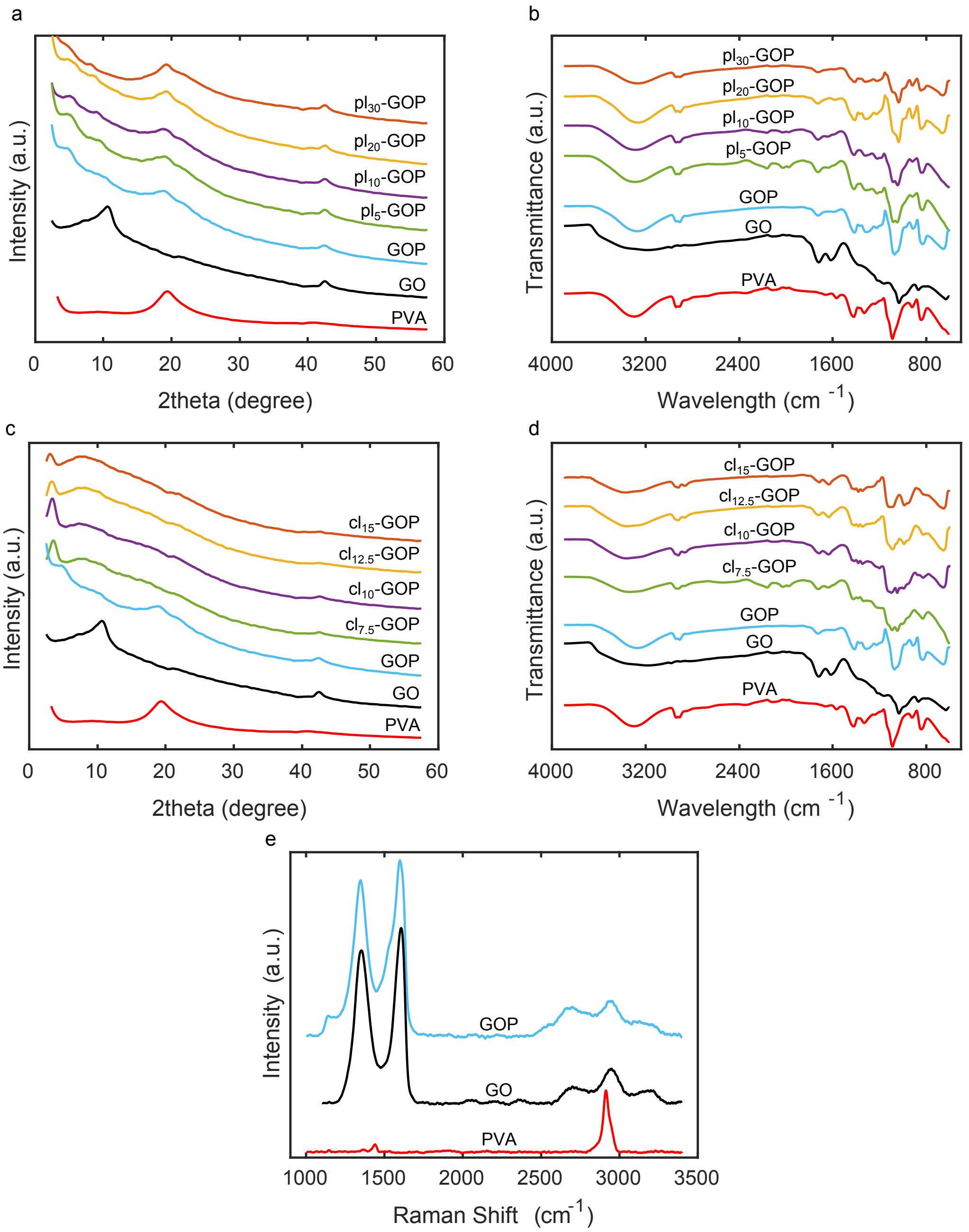

Figure S2. Physicochemical characterization of modified GOP aerogels. (a, c) XRD patterns and **(b, d)** FT-IR spectra of **(a, b)** pl$_x$-GOP and **(c, d)** cl$_y$-GOP with pure GO and PVA as reference. **(e)** Raman spectra of un-modified GOP with pure GO and PVA as reference.

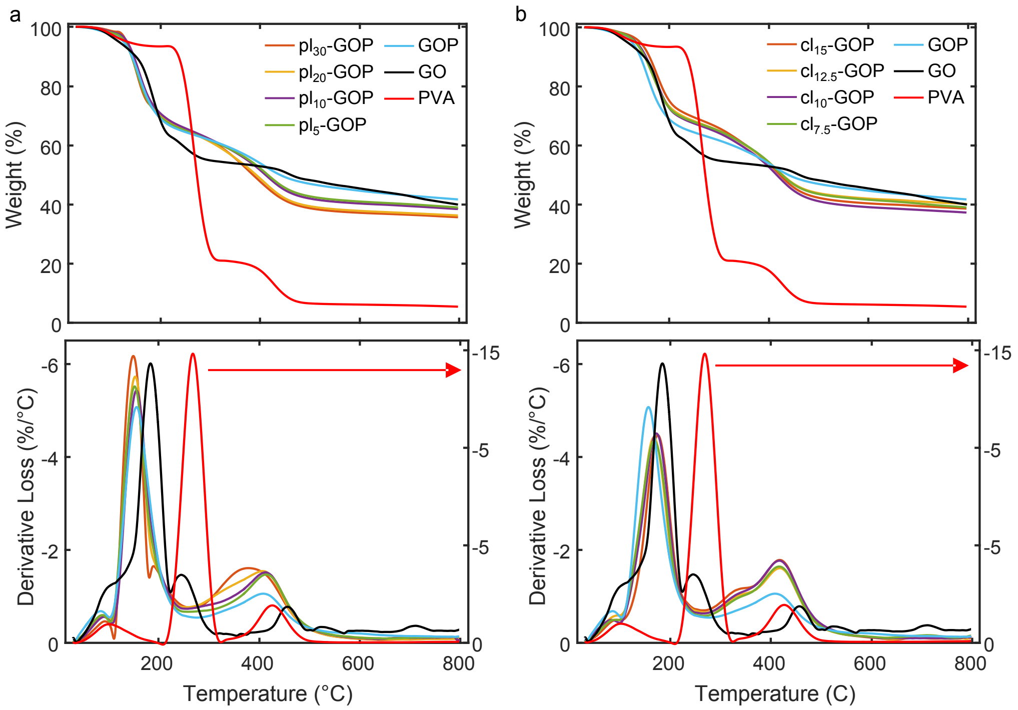

Figure S3. Thermal stability of modified GOP aerogels. (a,b) TGA and **(c,d)** dTGA of **(a,c)** pl$_x$-GOP and **(b,d)** cl$_y$-GOP aerogels.

2. Supporting tables

Table S1. Comparison of density, porosity, sound absorption and thermal conductivity between modified GOP aerogels from this work and other aerogels with comparable thickness previously reported in the literature.

[A] The average was calculated in the 500 – 1500 Hz range.

Name	Density (kg m^{-3})	Thickness (mm)	Average[A] Absorption Coefficient	Thermal conductivity (W mK^{-1})	Ref.
Mod. GOP	3.65 – 19.92	25	0.54 – 0.72	0.0391	This work
GOP	2.10	25	0.68	0.0374	This work, [1]
BGM	2.6 – 10.3	30	0.33 – 0.74 *[800-1500 Hz]*	-	[2]
MFGO	12.4 – 24.1	26	0.40 – 0.69	-	[3]
GONPVA	-	20	0.71	0.0255	[4]
MTES	53 – 70	12	0.226	0.022 – 0.026	[5]
RA	57 – 126	30	0.43 – 0.55	0.035 – 0.049	[6]
Lignin Aerogels	24 – 80	-	0.47 – 0.77	0.128 – 0.174	[7]
CNFAs	0.5 – 10	15	-	0.025 – 0.032	[8]
PVA/CNF/ MWCNT	20.1 – 30.6	-	-	0.028 – 0.030	[9]
CAP	20 – 70	20	0.57	0.031	[10]
NRA	25	30	0.61	0.035	[11]
BRAA	11	30	0.60	0.016	[12]

References

1. Rapisarda, M.; Malfense Fierro, G.-P.; Meo, M., Ultralight graphene oxide/polyvinyl alcohol aerogel for broadband and tuneable acoustic properties. *Scientific Reports* **2021,** *11* (1), 10572.

2. Lu, B.; Lv, L.; Yang, H.; Gao, J.; Xu, T.; Sun, G.; Jin, X.; Shao, C.; Qu, L.; Yang, J., High performance broadband acoustic absorption and sound sensing of a bubbled graphene monolith. *Journal of Materials Chemistry A* **2019,** *7* (18), 11423-11429.

3. Nine, M. J.; Ayub, M.; Zander, A. C.; Tran, D. N. H.; Cazzolato, B. S.; Losic, D., Graphene Oxide-Based Lamella Network for Enhanced Sound Absorption. *Advanced Functional Materials* **2017,** *27* (46), 1703820.

4. Simón-Herrero, C.; Peco, N.; Romero, A.; Valverde, J. L.; Sánchez-Silva, L., PVA/nanoclay/graphene oxide aerogels with enhanced sound absorption properties. *Applied Acoustics* **2019,** *156*, 40-45.

5. Li, X.; Yang, Z.; Li, K.; Zhao, S.; Fei, Z.; Zhang, Z., A flexible silica aerogel with good thermal and acoustic insulation prepared via water solvent system. *Journal of Sol-Gel Science and Technology* **2019,** *92* (3), 652-661.

6. Thai, Q. B.; Chong, R. O.; Nguyen, P. T. T.; Le, D. K.; Le, P. K.; Phan-Thien, N.; Duong, H. M., Recycling of waste tire fibers into advanced aerogels for thermal insulation and sound absorption applications. *Journal of Environmental Chemical Engineering* **2020,** *8* (5), 104279.

7. Wang, C.; Xiong, Y.; Fan, B.; Yao, Q.; Wang, H.; Jin, C.; Sun, Q., Cellulose as an adhesion agent for the synthesis of lignin aerogel with strong mechanical performance, Sound-absorption and thermal Insulation. *Scientific Reports* **2016,** *6* (1), 32383.

8. Si, Y.; Wang, X.; Dou, L.; Yu, J.; Ding, B., Ultralight and fire-resistant ceramic nanofibrous aerogels with temperature-invariant superelasticity. *Science Advances* **2018,** *4* (4), eaas8925.

9. Zheng, Q.; Javadi, A.; Sabo, R.; Cai, Z.; Gong, S., Polyvinyl alcohol (PVA)–cellulose nanofibril (CNF)–multiwalled carbon nanotube (MWCNT) hybrid organic aerogels with superior mechanical properties. *RSC Advances* **2013,** *3* (43), 20816-20823.

10. Pornea, A. G. M.; Puguan, J. M. C.; Ruello, J. L. A.; Kim, H., Multifunctional Dual-Pore Network Aerogel Composite Material for Broadband Sound Absorption, Thermal Insulation, and Fire Repellent Applications. *ACS Applied Polymer Materials* **2022,** *4* (4), 2880-2895.

11. Thai, Q. B.; Le-Cao, K.; Nguyen, P. T. T.; Le, P. K.; Phan-Thien, N.; Duong, H. M., Fabrication and optimization of multifunctional nanoporous aerogels using recycled textile fibers from car tire wastes for oil-spill cleaning, heat-insulating and sound absorbing applications. *Colloids and Surfaces A: Physicochemical and Engineering Aspects* **2021,** *628*, 127363.

12. Nie, Z.-J.; Wang, J.-X.; Huang, C.-Y.; Feng, J.-F.; Fan, S.-T.; Tan, M.; Yang, C.; Li, B.-J.; Zhang, S., Hierarchically and wood-like cyclodextrin aerogels with enhanced thermal insulation and wide spectrum acoustic absorption. *Chemical Engineering Journal* **2022,** *446*, 137280.
